# Comparative Phytochemical Profile, Antioxidant, Antimicrobial and *In Vivo* Anti-Inflammatory Activity of Different Extracts of Traditionally Used Romanian *Ajuga genevensis* L. and *A. reptans* L. (Lamiaceae)

**DOI:** 10.3390/molecules24081597

**Published:** 2019-04-23

**Authors:** Anca Toiu, Andrei Mocan, Laurian Vlase, Alina Elena Pârvu, Dan Cristian Vodnar, Ana-Maria Gheldiu, Cadmiel Moldovan, Ilioara Oniga

**Affiliations:** 1 Department of Pharmacognosy, “Iuliu Hațieganu” University of Medicine and Pharmacy, 400337 Cluj-Napoca, Romania; ancamaria_toiu@yahoo.com (A.T.); ioniga@umfcluj.ro (I.O.); 2 Department of Pharmaceutical Botany, “Iuliu Hațieganu” University of Medicine and Pharmacy, 400337 Cluj-Napoca, Romania; mocan.andrei@umfcluj.ro (A.M.); Gheldiu.Ana@umfcluj.ro (A.-M.G.); moldovan.cadmiel@yahoo.com (C.M.); 3 Department of Pharmaceutical Technology and Biopharmacy, “Iuliu Hațieganu” University of Medicine and Pharmacy, 400337 Cluj-Napoca, Romania; 4 Department of Pathophysiology, “Iuliu Hațieganu” University of Medicine and Pharmacy, 400337 Cluj-Napoca, Romania; parvualinaelena@yahoo.com; 5 Department of Food Science, Faculty of Food Science and Technology, University of Agricultural Sciences and Veterinary Medicine, 400372 Cluj-Napoca, Romania; dan.vodnar@usamvcluj.ro

**Keywords:** *Ajuga genevensis*, *A. reptans*, polyphenols, iridoids, sterols, anti-inflammatory

## Abstract

Several *Ajuga* species are used in Romanian folk medicine for their antioxidant, antimicrobial and anti-inflammatory properties, to treat pain, fever or arthritis. Still, the active compounds responsible for these effects and their mechanism of action are scarcely known. This research was designed to investigate the phytochemical profile (e.g. iridoids, polyphenolic compounds, phytosterols), as well as the biological potential (antioxidant, antibacterial, antifungal, anti-inflammatory properties) of two selected *Ajuga* species collected from different regions of Romanian spontaneous flora. The main compounds identified in *A. reptans* aerial parts extracts were 8-*O*-acetylharpagide, isoquercitrin and β-sitosterol, whilst in *A. genevensis* were 8-*O*-acetylharpagide, luteolin and campesterol. The extracts were screened for their antioxidant potential using different methods (DPPH, TEAC, EPR) and the results showed a good activity, in accordance with the polyphenol content (18–26 mg GAE/g dw). The antifungal activity on the tested strains was good. The determination of few parameters linked with the inflammatory mechanism allowed the assessment of in vivo anti-inflammatory potential. *Ajuga reptans* and *A. genevensis* ethanol extracts had anti-inflammatory activity through lowering the oxidative stress, phagocytosis, PMN and total leukocytes. The best anti-oxidative and anti-inflammatory activity was observed for the *Ajuga reptans* 100 mg dw/mL extract when compared with diclofenac, thus the dose could be correlated with the pharmacological effect. These findings provide substantial evidence that both selected *Ajuga* species have the potential to be valued as sources of phytochemicals in effective anti-inflammatory herbal preparations.

## 1. Introduction

Over the last decades, medicinal plants have historically proved to be a valuable source of drug development candidates due to their important reserve of bioactive compounds [1]. In order to obtain new herbal medicines with important pharmacological properties, the secondary metabolites found in plants are excellent candidates. For example, omnipresent in vegetal products and important component of human nutrition, phenolics with antioxidant effects are of major interest for their valuable promising pharmacological properties [2]. Yet, numerous herbal products are empirically employed with no accurate evidence of therapeutic effects or lack of toxicity and usually their traditional importance is considered an evidence of safety and efficiency. Phytochemicals are biomolecules formed in plants, that occur in herbal drugs or phytopharmaceuticals, bioactive compounds which could lower the risk of certain disorders [3]. In this context, in order to obtain new herbal preparations, additional investigations into unexplored herbal products used in phytotherapy are required. 

Phytosterols are found in medicinal and edible plants and campesterol, *β*-Sitosterol and stigmasterol prevail in the class of active compounds. They are also present in numerous vegetables, seeds and their oils [4]. Several studies showed the beneficial effects of sterols from plants on serum cholesterol levels [5,6]. The well-documented potency of phytosterols in decreasing serum cholesterol levels has led to the development of phytosterol-enriched herbal drugs or dietary supplements. On the other hand, research interest in the role of phytosterols at dietary levels achieved by consuming food items (herbal preparations, dietary supplements) which are naturally rich in phytosterols, is also increasing [7]. 

Fifty species and about 300 taxa are included in *Ajuga* genus from Lamiaceae. Many species are widespread in Asia, Europe or Africa. The herbaceous flowering plants have opposite leaves and 5–50 cm tall [8,9]. Many *Ajuga* species have been used as a traditional medicine for the treatment of inflammation, pain, diabetes, hypertension or gastrointestinal disorders [10]. Recent studies have demonstrated their efficacy as anti-inflammatory agents [11,12], antioxidant, cytotoxic [13], antimalarial [10], hypolipidemic [14], analgesic [15,16], anabolic, antibacterial, antifungal, cardiotonic, hepatoprotective agents [17]. It has been reported that many species of *Ajuga* are rich in diterpenes, iridoids, flavonoids, anthocyanins, ecdysteroids, essential oils [12,13,18,19,20,21]. 

*Ajuga genevensis* L. (blue bugle, blue bugleweed or Geneva bugleweed) is a pubescent plant widespread in many countries of Europe. “Suliman” is the Romanian name of the species and traditionally is used for sedative, antihemorrhagic and anti-inflammatory effects, as well as for its wound-healing and epithelization capacity in topic remedies [22]. Also, the plant is used for its remarkable property to precipitate the proteins from digestive tract, in treatment of diarrhoeal diseases. 

*Ajuga reptans* L. (bugleweed, bugle or common bugle) is a flowering plant native to Europe. “Vineriţă” is the Romanian name of the species and the antioxidant and antimicrobial properties shown previously are linked with the presence of polyphenolic or iridoidic compounds [23].

In this regard, continuing our research on traditionally used *Ajuga* species [12], comparative phytochemical determinations of polyphenols, phytosterols and iridoids from *Ajuga genevensis* and *A. reptans* aerial parts extracts, as well as the evaluation of their antioxidant, antimicrobial and *in vivo* anti-inflammatory effects were performed. 

## 2. Results and Discussion

### 2.1. The Quantification of Total Bioactive Compounds 

Methanol and ethanol have been proven as effective solvents for extraction of phenolic compounds. As they have quite similar polarity, the bioactive compounds extracted in these solvents are very similar, therefore the detected differences between the methanol and ethanol extracts were only quantitatively ones. The main reason for this concept was that usually, the methanol extract is obtained for the purpose of phytochemical analysis, in laboratory, thus a rapid and simple extraction method is useful for the characterization of vegetal products (30 min at 60 °C), whereas the extract used in phytotherapy is generally the ethanol one, obtained by maceration at room temperature (7 days by maceration). Taking this into account, we intended to compare the results obtained by the two methods in order to evaluate if the theoretical method (with methanol) will extract the same compounds and in similar concentration and if those results could be extrapolated into practice, to ensure a more practical approach of our study.

*Ajuga* species contain a broad spectrum of phenolic compounds and many studies pointed out that these compounds may enhance the antioxidant activity of extracts. A comparation between the total phenolic, flavonoid and iridoid contents (TPC, TFC and TIC) of *Ajuga genevensis* (*Ag1, Ag2, Ag3*) and *A. reptans* (*Ar1, Ar2, Ar3*) different extracts (EE, ME) are presented in Table 1. The highest concentration of phenolics was obtained for the earliest collected samples of *A. genevensis* (22.59 ± 0.75 mg GAE/g dw in methanol extract and 26.78 ± 0.84 mg GAE/g dw, in ethanol extract). Interestingly, for both species and for both extracts, the TPC values decreased to the lowest concentration in the next month (*Ag2, Ar2*) and afterwards, the TPC values increased again (*Ag3, Ar3*). The variation in phenolic amounts could also be due to the status of different secondary metabolites in different growing locations, to genetic factors or ecological ones, as shown previously [24,25]. The obtained results regarding TPC values of *A. reptans* are comparable with the ones provided [23]: the methanol and ethanol flower extracts contain an amount of 20.86 ± 0.53, 24.11 ± 0.57 mg GAE/g respectively. Another research evaluating the chemical composition of aerial parts from *Ajuga chamaecistus* subsp. *scoparia* Rech.f. found 20.32 ± 0.39 mg GAE/g (meaning 2.64 mg GAE/g dw), which is lower than the *Ajuga* species considered herein [20]. 

A comparable trend was detected for the total flavonoid content (TFC) for ethanol and methanol extracts of evaluated *Ajuga* species. The highest values were obtained for *Ajuga sp.* harvested in the first stage of sample collection (*Ag1*) (15.91 ± 0.78 and 18.72 ± 0.85 mg RE/g dw). These values are slightly lower than the ones presented by Jakovljević et al., 2015 for the methanol, acetone and ethyl acetate extracts from *Ajuga chamaepitys* (L.) Schreb (61.77 ± 0.51, 63.87 ± 0.66 and 91.76 ± 0.81 mg RE/g, respectively). In a previous study, the total flavonoid content for the flowers of *A. reptans* methanol extract was evaluated. The results were 12.38 ± 0.22 mg RE/g dw [23], which is in accordance with the current results (11.26 ± 0.58 and 14.05 ± 0.41 mg RE/g dw). A recent study performed by Rani et al. (2017) revealed a TFC value of 9.3 mg QE/g dw for *Ajuga bracteosa* [26].

Considering the total iridoid content of various *Ajuga* species, there is limited accessible information. Precedent study [23] showed that *Ajuga reptans* ethanol extract from flowers contains iridoids in higher concentration, compared to the values presented herein (27.49 ± 0.94 vs. 21.03 ± 0.99 mg AE/g dw). This could be explained by the differences regarding active compounds from vegetal material, as several research observed higher bioactive compounds content in leaf extract than stem extracts [27,28,29]. Interestingly, higher TIC values were obtained for *A. reptans* than for *A. genevesis* methanol and ethanol extracts. Iridoids are known as active compounds with important biological activities. They could provide protection against various chemical, physical or biological stressors. Nonetheless, these compounds could provide a normalizing effect without affecting the physiologically functioning systems of human body [30].

### 2.2. Qualitative and Quantitative Analysis of Polyphenols 

In order to analyse the phenolic compounds from *Ajuga genevensis* (*Ag1*) and *A. reptans* (*Ar1*) extracts, an improved HPLC/UV/MS method was employed, using 18 polyphenolic compounds as standards. The aerial parts extracts from the two selected *Ajuga* species contain hyperoside, isoquercitrin, rutin and quercitrin (flavonoid glycosides), caffeic, *p*-coumaric and ferulic acids (phenolcarboxylic acids), apigenin and luteolin (free aglycones).

Table 2 summarizes the content in polyphenols in *Ajuga genevensis* and *A. reptans* extracts, expressed as µg/g dw.

Isoquercitrin—known as quercetin-3-glucoside—was the main polyphenol from *A. reptans* extracts (180.77 ± 2.84 and 151.1 ± 2.77 μg/g dw, respectively). The positive biological activity of isoquercitrin was assessed in vitro and in vivo, this compound being known to exert chemoprotective activities against allergic reactions, oxidative stress, diabetes, cardiovascular disorders and cancer. Current research on isoquercitrin indicates that it is more water-soluble and bioavailable than its aglycone. After oral administration, isoquercitrin can be detected unmodified in plasma and tissues [31]. Interestingly, the compound was not found in *A. genevensis* extracts at all. Five polyphenolic compounds were identified in all extracts (*p*-coumaric, ferulic acids, quercitrin, luteolin and apigenin), two phenolcarboxylic acids only in *A. genevensis* extracts, whilst two flavonoid glycosides only in *A. reptans* extracts. The pattern of polyphenols could be employed as potential taxonomic markers due to the fact that important differences between the two *Ajuga* species were observed.

### 2.3. Qualitative and Quantitative Analysis of Phytosterolic Compounds

Concentrations of phytosterols in the *Ajuga genevensis* (*Ag1*) and *A. reptans* (*Ar1*) extracts (EE, PEE, CE) are presented in Table 3. Due to the fact that three compounds (ergosterol, stigmasterol, brassicasterol) were found in very small amount (below the limit of quantification), they were only identified in all extracts. Significant differences between the analysed *Ajuga genevensis* extracts were observed: campesterol was identified in PEE and CE, with higher amounts in the latter. *A. reptans* CE was richer in phytosterols and the main compound was *β*-sitosterol (10923.02 ± 18.65 μg/mL), while a significant smaller content was determined in EE (2048.28 ± 9.31 μg/mL). The results can be correlated with previous studies regarding the two analysed *Ajuga* species. Stigmasterol and *β*-sitosterol were identified by TLC in both *Ajuga* species extracts by Ghita et al. (2011) [32]. *Ajuga relicta, A. bracteosa* and *A. taiwanensis* extracts contain these compounds, stating once more that those compounds are common in *Ajuga* genus [17]. 

### 2.4. Qualitative and Quantitative Analysis of Iridoids

The iridoids are considered chemotaxonomical markers in *Ajuga* species and various studies identified iridoid glycosides such as harpagide in several *Ajuga* plants [33,34]. Previous research demonstrated the anti-inflammatory, diuretic, antipyretic and astringent properties of some *Ajuga* species correlated with the presence of iridoids [12,35,36]. 

In the present study, the HPLC-MS/MS analysis of iridoids from *A. genevensis* (*Ag1, Ag2, Ag3*) and *A. reptans* (*Ar1, Ar2, Ar3*) aerial parts extracts was performed. From the obtained results (Table 4), it might be noticed that the major compound identified in all extracts was 8-*O*-acetylharpagide (481.2 ± 5.76, 470.6 ± 5.44 and 462.5 ± 5.23 μg/mL in *Ag1, Ag2* and *Ag3* ethanol extracts, respectively), followed by harpagide (199.7 ± 4.92, 186.4 ± 3.71 and 180.7 ± 3.5 μg/mL in *Ag1, Ag2* and *Ag3* ethanol extracts), while the aucubin and catalpol were found in lower amounts. The important content in iridoids could be directly connected with their pharmaceutical effectiveness. In all analysed samples, we observed that ethanol extracts contain higher amounts of iridoids than methanol extracts and that is in accordance with preceding research [12]. Several iridoids (namely ajugoside, reptoside, 8-*O*-acetylharpagide, harpagide) which are known as markers for various *Lamiaceae* species were isolated from *A. chamaepitys* aerial parts from Italy in a former study [13]. The determination of iridoids in *A. genevensis* and *A. reptans* extracts using the simple, rapid and accurate HPLC-MS/MS method allowed the standardization of these extracts. The HPLC analysis revealed that *A. reptans* aerial parts contain the highest amount of iridoids and the spectrophotometric determinations showed the same tendency. The high content in iridoid glycosides could explain the antifungal and anti-inflammatory effects of *Ajuga* sp. extracts, in accordance with previous studies [12,36,37]. 

The phytochemical evaluation of the six samples collected in different harvesting time and locations showed that the aerial parts of *A. genevensis* and *A. reptans* harvested in April from Cluj County contain the highest number of polyphenolic compounds and iridoids, respectively.

The obtained results allow the characterization of *A. genevensis* and *A. reptans* aerial parts extracts in main biologically active compounds, therefore the possibility of correlation between the therapeutic effect and the effective dose. 

### 2.5. The Evaluation of Antioxidant Activity

#### 2.5.1. DPPH (2,2-diphenyl-1-picrylhydrazyl) assay and ABTS (2,2’-azinobis-(3-ethylbenzothiazoline-6-sulfonic acid)) Radical Scavenging Activity

Considering the presented results for phytochemical characterization of *A. genevensis* (*Ag1, Ag2, Ag3*) and *A. reptans* (*Ar1, Ar2, Ar3*) aerial parts, the evaluation of bioactivities was performed on the plant materials with higher content in active compounds. Therefore, the antioxidant activity of *A. genevensis* (*Ag1*) and *A. reptans* (*Ar1*) was evaluated for methanol and ethanol extracts, the results being summarized in Table 5 (TEAC—Trolox equivalent antioxidant capacity, EPR—electron paramagnetic resonance spectroscopy). The best DPPH IC_50_ values were obtained for *A. genevensis* methanol and ethanol extracts: 33.74 ± 1.99 and 31.29 ± 1.92 μg/mL, which is considered a high antioxidant effect with an IC_50_ ≤ 50 μg/mL [38]. Compared with other studies, all extracts exerted a higher antiradical activity. Another study documented the antioxidant effect for *A. turkestanica* roots extract (57.84 ± 4.19 μg/mL) [33], while another study concerning *Ajuga reptans* reported a bigger amount (65.7 ± 3.82 μg/mL) [21]. A previous study focused on the methanol and ethanol flower extract of *A. genevensis* showed IC_50_ values of 72.08 ± 6.02 and 45.45 ± 3.27 µg/mL, respectively [22].

ABTS is a free radical which is currently employed for the assessment of antioxidant effects. Considering the TEAC method, the values of antioxidant activities for *Ajuga genevensis* (*Ag1*) and *A. reptans* (*Ar1*) ethanol extracts were 66.13 ± 2.87 and 60.98 ± 1.52 mg TE/g extract (presented in Table 5), lower than for the previous studied *Ajuga laxmannii* species (71.07 ± 2.40 mg TE/g extract) [12]. The high antioxidant capacity of *Ajuga genevensis* and *A. reptans* could be correlated with the content in polyphenolic compounds, found in higher concentration in *A. genevensis* aerial parts extracts. 

#### 2.5.2. Electron Paramagnetic Resonance Spectroscopy (EPR)

**Figure 1:**
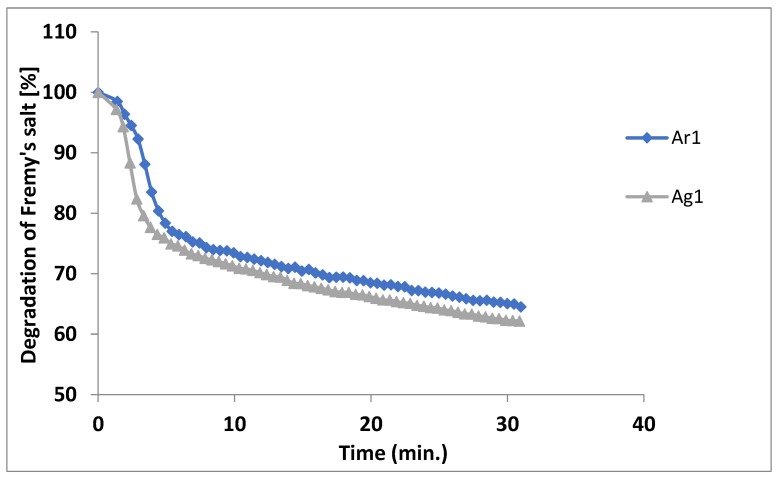
EPR measurements: *Ag1*—*A. genevensis* extract, *Ar1*—*A. reptans* extract.

In order to correlate the results obtained with the ones from TEAC method, the *A. genevensis* (*Ag1*) and *A. reptans* (*Ar1*) ethanol extracts were further analysed by EPR method. Potassium nitrosodisulfonate (Fremy’s salt, FS) was used as a stable radical. The method quantified the degradation of the stable radical in the presence of antioxidants from the *Ajuga* species extracts. After 30 min, an amount of 94.915 ± 5.24 and 88.896 ± 4.01 mg FS/g extract was determined for *Ajuga genevensis* and *A. reptans*, respectively (Table 5). The degradation of the salt is ~80% for both extracts after 5 min of incubation, with a slightly higher value for *A. reptans*. Moreover, the time interval from 5 to 30 min, the degradation of Fremy’s salt is quite constant and after the interval considered, the value is ~70%. Figure 1 summarizes the Fremy’s salt’s kinetic degradation.

The EPR is a properly described, common, recognized method for the evaluation of the free radicals kinetic degradation. In a previous study regarding *A. laxmannii* extracts, the EPR value was slightly higher, of 98.073 ± 1.23 mg FS/g dw [12].

### 2.6. The Evaluation of Antibacterial Activity

Different Gram− and Gram+ bacteria strains were employed for estimation of *Ajuga* species extracts antibacterial effects. The antimicrobial activity was determined using gentamycin as standard, by microdilution assay. Minimum Inhibitory Concentration (MIC) and Minimum Bactericidal Concentration (MBC) were calculated and expressed as mg extract/mL.

The values of MIC obtained for the *Ajuga genevensis* were between 0.78–6.25 mg/mL for EE and 1.56–6.25 mg/mL for ME (Table 6). Regarding the *Ajuga reptans* extracts, the MIC values were comparable with those obtained for *A. genevensis* extracts, the only notable difference being assessed for ethanol extract, with values from 0.78 to 3.12 mg/mL. All extracts showed comparable activities against four bacterial strains: *Escherichia coli, Listeria monocytogenes, Pseudomonas aeruginosa and Salmonella typhimurium*. *Ajuga genevensis* and *A. reptans* ethanol extracts exerted the best antimicrobial activity against *S. aureus* with MIC = 0.78 mg/mL, MBC = 1.56 mg/mL for both species. *Escherichia coli*, *Salmonella typhimurium and Listeria monocytogenes* were the less susceptible strains for methanol extracts obtained from both species, with a MBC value of 12.5 mg/mL. A previous study [23] showed a comparable tendency for *Ajuga reptans* flower extracts. Good antibacterial effects can be correlated with MIC values around or less 0.5 mg/mL [39]. Therefore, modest antibacterial effects for *Ajuga genevensis* and *A. reptans* aerial parts extracts were determined. 

In general, the ethanol extracts of *Ajuga* species exerted the highest activity against the bacterial strains tested. Some conclusions can be drawn taking into account the HPLC–MS results presented herein, concerning the antibacterial capacity of *Ajuga sp.* extracts. The ethanol extracts contain higher amounts of polyphenols than the methanol ones. Recently, many studies have focused on the potential of the phenolic compounds to exert antibacterial activity [40,41]. The presence of phenolics in ethanol extracts could determine the antimicrobial capacity exerted by *Ajuga* species.

### 2.7. The evaluation of Antifungal Activity

The extracts (EE, PEE, CE) of *Ajuga genevensis* (*Ag1*) and *A. reptans* (*Ar1*) were tested for their antifungal activity by using five different species of fungi, which were selected based on the importance in public health. The evidence of antifungal properties exerted by the extracts from *Ajuga genevensis* (*Ag1*), *A. reptans* (*Ar1*) used against tested strains is summarized in Table 7. The best susceptibility to the *Ajuga reptans* petroleum ether extract was shown by *Candida albicans* (0.006 mg/mL and 0.012 mg/mL for minimum inhibitory concentration (MIC) and minimum fungicidal concentration (MFC). From the same *Ajuga* species, following highest sensitivity was shown to chloroform and ethanol extracts, with MFC = 0.025 mg/mL and MIC = 0.012 mg/mL, respectively and the most resistant strains against ethanol extract of *Ajuga genevensis* being *Penicillium fumiculosum* and *Aspergillus niger* (MFC 0.2 mg/mL). In their article, Kawamura and Ohara, (2005) stated that high level of iridoids contained within the plant extracts might be linked to the antifungal activity of these extracts. All extracts of aerial parts from *Ajuga* species showed high concentrations of iridoid glycosides, mainly 8-*O*-acetylharpagide. This outcome is in conformity with studies that were performed previously [12], where the antifungal activity of *Ajuga laxmannii* aerial parts was determined.

### 2.8. The Evaluation of In Vivo Anti-Inflammatory Activity

Three *Ajuga genevensis* (*Ag1*) and *A. reptans* (*Ar1*) ethanol extracts (25 mg dw/mL, 50 mg dw/mL and 100 mg dw/mL) were tested for their anti-inflammatory effects in vivo. The experimental rat acute inflammation was induced by a non-antigenic inflammatory stimulus. The turpentine oil activates inflammatory cytokines, reactive oxygen species (ROS) and NO release. High serum concentrations of ROS and NOx are considered as positive markers of oxidative stress in the inflammatory response. Diclofenac was used as the positive control.

**Figure 2:**
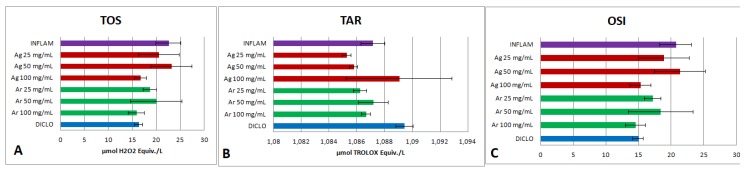
Oxidative stress tests. (**A**) Total oxidative status (TOS). (**B**) Total antioxidant response (TAR). (**C**) Oxidative stress index (OSI). Note: Diclo—animals were given 20 mg/kg BW diclofenac, Inflam—the induction of inflammation was made by intramuscular injection of turpentine oil (6 mL/kg BW), *Ag* 25 mg/mL—animals were given 5 mL/kg BW *A. genevensis* ethanol extract 25 mg dw/mL, *Ag* 50 mg/mL—animals were given 5 mL/kg BW *A. genevensis* ethanol extract 50 mg dw/mL, *Ag* 100 mg/mL—animals were given 5 mL/kg BW *A. genevensis* ethanol extract 100 mg dw/mL, *Ar* 25 mg/mL—animals were given 5 mL/kg BW *A. reptans* ethanol extract 25 mg dw/mL, *Ar* 50 mg/mL—animals were given 5 mL/kg BW *A. reptans* ethanol extract 50 mg dw/mL, *Ar* 100 mg/mL—animals were given 5 mL/kg BW *A. reptans* ethanol extract 100 mg dw/mL (*p* < 0.001).

Oxidative stress may be the consequence of excessive ROS formation or of the antioxidants deficiency. For ROS synthesis evaluation was used the TOS. TOS was significantly increased (*p* < 0.001) by the inflammation induced by turpentine, whereas the diclofenac administration determined the decrease of this parameter significantly (*p* < 0.001) (Figure 2A). Remarkably, the administration of *Ajuga reptans* extract dilutions lowered TOS. The 25 mg dw/mL (*p* < 0.01) and 100 mg dw/mL (*p* < 0.001) extracts had the best inhibitory activities. The treatment with diclofenac had a comparable effect on TOS inhibition (*p* < 0.01), in comparison with the administration of 100 mg dw/mL (*p* < 0.01) and 25 mg dw/mL (*p* < 0.01) extracts. The *Ajuga reptans* and *A. genevensis* 50 mg dw/mL extracts did not influence TOS significantly (*p* > 0.05). 

For antioxidant defence mechanism, TAR was evaluated. TAR was slightly increased by the diclofenac treatment (*p* < 0.05) and was reduced for inflammation group (*p* < 0.05) (Figure 2B). *Ajuga genevensis* 100 mg dw/mL extract increased TAR (*p* < 0.01) and the effect was as good as that of diclofenac (*p* > 0.05). 

The general evaluation of the oxidative stress was performed using the OSI. The parameter was significantly elevated in the inflammation group (*p* < 0.001), whilst a decrease of OSI was observed for diclofenac treatment (*p* < 0.001) (Figure 2C). The *Ajuga reptans* 100 mg dw/mL extract and *A. genevensis* 100 mg dw/mL extracts induced an important reduction of OSI (*p* < 0.001), comparable with the diclofenac effect (*p* < 0.05). 

**Figure 3:**
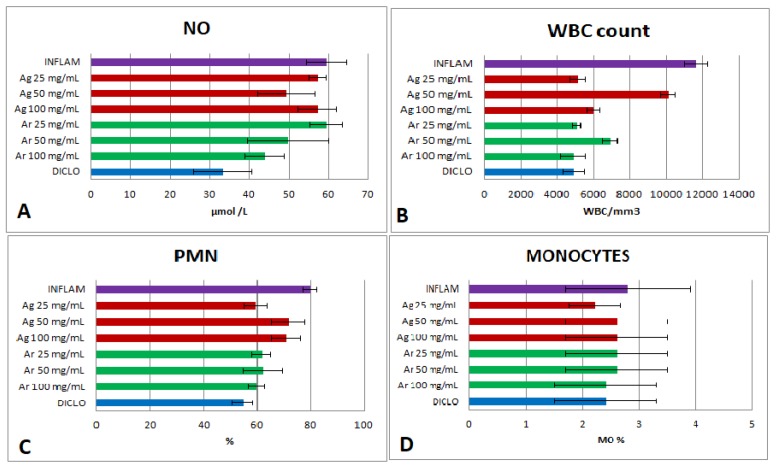
Anti-inflammatory effect tests. (**A**) NO synthesis. (**B**) White blood cells (WBC) count. (**C**) Polymorphonuclear leukocytes (PMN). (**D**) Total number of monocytes (MO). Note: Diclo—animals were given 20 mg/kg BW diclofenac, Inflam—the induction of inflammation was made by intramuscular injection of turpentine oil (6 mL/kg BW), *Ag* 25 mg/mL—animals were given 5 mL/kg BW *A. genevensis* ethanol extract 25 mg dw/mL, *Ag* 50 mg/mL—animals were given 5 mL/kg BW *A. genevensis* ethanol extract 50 mg dw/mL, *Ag* 100 mg/mL—animals were given 5 mL/kg BW *A. genevensis* ethanol extract 100 mg dw/mL, *Ar* 25 mg/mL—animals were given 5 mL/kg BW *A. reptans* ethanol extract 25 mg dw/mL, *Ar* 50 mg/mL—animals were given 5 mL/kg BW *A. reptans* ethanol extract 50 mg dw/mL, *Ar* 100 mg/mL—animals were given 5 mL/kg BW *A. reptans* ethanol extract 100 mg dw/mL (*p* < 0.001).

ROS may react with NO generating reactive nitrogen species (RNS), which are also strong oxidants. NO synthesis was indirectly evaluated by measuring NOx. NOx was reduced in the group treated with diclofenac (*p* < 0.01), and, moreover was significantly higher in the inflammation group (*p* < 0.01) (Figure 3A). The *A. reptans* 100 mg dw/mL extract reduced NOx significantly (*p* < 0.01), compared with the inflammation group. There was no important inhibitory activity on NOx in all *A. genevensis* extracts (*p* > 0.05). The OSI was correlated with NOx (r = 0.81) and TOS (r = 0.92).

The evaluation of anti-inflammatory properties of *Ajuga* species extracts was also achieved by calculating the WBC count and the differential WBC count. All *Ajuga reptans* extracts reduced the total WBC count in a significant manner (*p* < 0.001) compared to the inflammation group (Figure 3B) by lowering PMN % (Figure 3C) and MO % (Figure 3D). *Ajuga reptans* 25 mg dw/mL and 100 mg dw/mL extracts were the best inhibitors (*p* < 0.001) on the WBC and they were similar to diclofenac effects (*p* > 0.05). 

*Ajuga genevensis* extracts reduced in a significant manner (*p* < 0.001) the total WBC count (Figure 3B) by reducing PMN % (Figure 3C) and MO % (Figure 3D), in comparison with the inflammation group. 

**Figure 4:**
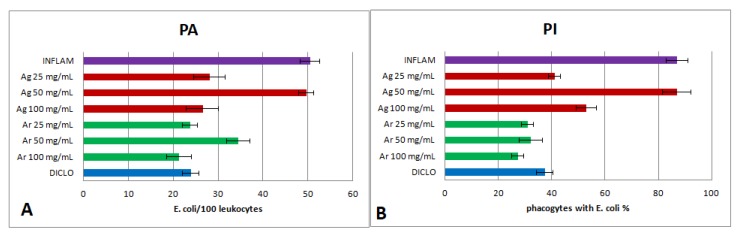
Phagocytosis test parameters. (**A**) Phagocytic activity (PA). (**B**) Phagocytic index (PI). Note: Diclo—animals were given 20 mg/kg BW diclofenac, Inflam—the induction of inflammation was made by intramuscular injection of turpentine oil (6 mL/kg BW), *Ag* 25 mg/mL—animals were given 5 mL/kg BW *A. genevensis* ethanol extract 25 mg dw/mL, *Ag* 50 mg/mL—animals were given 5 mL/kg BW *A. genevensis* ethanol extract 50 mg dw/mL, *Ag* 100 mg/mL—animals were given 5 mL/kg BW *A. genevensis* ethanol extract 100 mg dw/mL, *Ar* 25 mg/mL—animals were given 5 mL/kg BW *A. reptans* ethanol extract 25 mg dw/mL, *Ar* 50 mg/mL—animals were given 5 mL/kg BW *A. reptans* ethanol extract 50 mg dw/mL, *Ar* 100 mg/mL—animals were given 5 mL/kg BW *A. reptans* ethanol extract 100 mg dw/mL (*p* < 0.001).

Each extract dilution was then assessed for the capacity to inhibit phagocytosis in vitro. The *Ajuga genevensis* 25 mg dw/mL and *A. reptans* 100 mg dw/mL extracts exhibited the most important inhibitory activity towards phagocytosis. The effect of *Ajuga reptans* extract was correlated with a reduction of PA and PI (Figure 4A,B) (*p* < 0.001). *Ajuga genevensis* 25 mg dw/mL and 100 mg dw/mL extracts also determined a reduction of PI (Figure 4B) (*p* < 0.001). 

The presented results are in accordance with the hypothesis that extracts from *Ajuga genevensis* (*Ag1*) and *A. reptans* (*Ar1*) aerial parts have anti-inflammatory effects through the inhibition of phagocytosis by the reduction of oxidative stress (Table 8).

*Ajuga reptans* and *A. genevensis* extracts exerted a lower anti-nitro-oxidative stress and anti-inflammatory effect than treatment with diclofenac (*p* < 0.001). *Ajuga reptans* 100 mg dw/mL extract exerted the best effect compared to diclofenac.

Previous studies suggest that some natural products may play an important anti-inflammatory role, because they inhibit NO synthesis. In the present research, only *A. reptans* 100 mg dw/mL extract proved to have important anti-inflammatory effects by reducing NO synthesis and it was comparable with that induced by diclofenac. Because the effects on NOx were not always correlated with the in vitro antioxidant tests, it may be presumed that the antioxidant activity was not significantly involved. In some human diseases, antioxidant therapy failure was called the antioxidant paradox. Due to the fact that overproduction of ROS could determine an inflammatory response and inflammatory mediators may induce an oxidative stress, it was generally accepted that oxidation and inflammation are interlinked processes. The latest explanation of antioxidant therapy failure comes from the finding that antioxidants do not inhibit oxidative stress and the associated inflammation at the same time [42,43].

As our results revealed, although the two *Ajuga* species extracts had comparable amounts of phytochemicals and close related results for in vitro evaluation of antioxidant effects were observed, in vivo studies showed significant differences in anti-inflammatory activities of three doses of the same (ethanol) extract. 

Whereas numerous in vitro and in vivo studies demonstrated the anti-inflammatory, antioxidant, neuroprotective effects of polyphenolic compounds, other research also highlighted the fact that rutin and quercetin derivatives can act as pro-oxidant molecules, depending on concentration and reaction conditions [44]. Therefore, it could be a dose dependent “double-edged sword” effect: lower doses have a moderate anti-oxidant effect, while higher doses could induce the increase of the levels of endogenous antioxidants and melanin production as an adaptive response to oxidative damage [45,46]. 

Consecutive, the use of ethanol extract in phytotherapy for anti-inflammatory effects requires the standardization in main active compounds in order to provide an effective herbal preparation.

## 3. Materials and Methods

### 3.1. Reagents and Chemicals 

Caffeic, chlorogenic, *p*-coumaric, gallic acids, isoquercitrin, rutin, quercetin, hyperoside, fisetin, quercetol, kaempferol, apigenin, myricetol, harpagoside, catalpol, aucubin, ergosterol, β-sitosterol, stigmasterol, brassicasterol, campesterol were standards from Merck (Darmstadt, Germany), 8-*O*-acetyl-harpagide, harpagide from PhytoLab (Vestenbergsgreuth, Germany), caftaric acid from Dalton (Toronto, ON, Canada), gentisic, sinapic, ferulic acids, luteolin, patuletin were obtained from Roth (Karlsruhe, Germany). Copper (II) sulphate pentahydrate, sodium carbonate, sodium acetate trihydrate, anhydrous aluminium chloride from Merck (Darmstadt, Germany). Solvents used for extraction and separation were HPLC analytical-grade (methanol, ammonium acetate, acetonitrile) or analytical-grade (acetic acid, hydrochloric acid, potassium hydroxide, petroleum ether, silver nitrate, *n*-hexane, chloroform) and Folin-Ciocâlteu reagent were acquired from Merck (Darmstadt, Germany). Fremy’s salt and horseradish peroxidase (HRP) purchased from Merck (Darmstadt, Germany), 2,2-diphenyl-1-picrylhydrazyl (DPPH) and 6-hydroxy-2,5,7,8-tetramethylchroman-2-carboxylic acid (*Trolox)* were from Alfa-Aesar (Karlsruhe, Germany).

### 3.2. Plant Material

Medicinal plants were harvested at full flowering stage from different areas and periods from Romanian spontaneous flora. The aerial parts of *Ajuga genevensis* harvested from Cluj County in April 2017 (*Ag1*), harvested from Alba County in May 2017 (*Ag2*), harvested from Neamţ County in June 2017 (*Ag3*), aerial parts of *Ajuga reptans* harvested from Cluj County in April 2017 (*Ar1*), harvested from Alba County in May 2017 (*Ar2*), harvested from Neamţ County in June 2017 (*Ar3*). Plants were identified and voucher samples were preserved in the Herbarium of Pharmacognosy Department, University of Medicine and Pharmacy, Cluj-Napoca.

The extraction of each sample was performed with 70% methanol, 30 minutes at 60 °C on a water-bath (methanol extract 10%, ME) and with 70% ethanol for 7 days by maceration (ethanol extract 10%, EE) [23]. Petroleum ether extracts 10% (PEE) and chloroform extracts 10% (CE) were obtained and used for the evaluation of antifungal properties, as well as for the identification and quantification of phytosterols [47]. 

### 3.3. Quantitative Analyses 

#### 3.3.1. Total Phenolics 

The determination of total phenolic content (TPC) of the extracts obtained from *Ajuga genevensis* (*Ag1, Ag2, Ag3*) and *A. reptans* (*Ar1, Ar2, Ar3*) aerial parts was carried out as reported in previous paper by Folin-Ciocâlteu method [48]. The content in total phenolics was expressed as mg gallic acid equivalents (GAEs)/g dry weight (dw) vegetal product. The experiments were performed in triplicate.

#### 3.3.2. Total Flavonoids 

The estimation of total flavonoid content (TFC) of the extracts obtained from *Ajuga genevensis* (*Ag1, Ag2, Ag3*) and *A. reptans* (*Ar1, Ar2, Ar3*) aerial parts was carried out using a spectrophotometric method [47]. The flavonoids content was expressed as rutin equivalents (mg REs)/g dw vegetal product. 

#### 3.3.3. Total Iridoids 

The assessment of total iridoid content (TIC) of the extracts obtained from *Ajuga genevensis* (*Ag1, Ag2, Ag3*) and *A. reptans* (*Ar1, Ar2, Ar3*) aerial parts was accomplished by a photometric method, by a Trim-Hill reaction. The content in total iridoids was expressed as aucubin equivalents (mg AEs)/g dw vegetal product [49]. 

### 3.4. The Evaluation of Antioxidant Activity

The assessment of the antiradical effect of *Ajuga genevensis* (*Ag1*) and *A. reptans* (*Ar1*) extracts was performed applying three methods: DPPH, ABTS radical scavenging and electron paramagnetic resonance (EPR) spectroscopy assays and described in previous papers [12,47,50]. The antioxidant capacity was expressed as IC_50_ (μg/mL) for DPPH and as Trolox equivalents (TEs)/g of extract for ABTS assay and the positive control used in antioxidant methods was Trolox.

An IC_50_ value less than 50 μg TEs/mL could indicate a very good antioxidant effect, the value between 50 and 100 μg TEs/mL might show a valuable antioxidant effect, between 100 and 200 μg TEs/mL could reveal a soft antioxidant effect, while a value greater than 200 μg TEs/mL could mean the lack of antioxidant effect [51].

The antioxidant effect of *Ajuga genevensis* (*Ag1*) and *A. reptans* (*Ar1*) ethanol extracts was also evaluated by electron paramagnetic resonance (EPR), as previously described [52,53]. A control reaction was used in order to calculate the antiradical potential of extracts (expressed as Fremy’s salt equivalents (FSE)/g dw). 

### 3.5. Qualitative and Quantitative Analysis of Polyphenols 

#### 3.5.1. Working Conditions–General Apparatus

For the identification and quantification of the polyphenolic compounds the following system was employed: Agilent 1100 HPLC Series (Agilent Technologies, Santa Clara, CA, USA), to which a mass spectrometer Agilent Ion Trap SL was coupled, the latter being equipped with an atmospheric pressure chemical ionization (APCI) ion source or electrospray ionization (ESI).

#### 3.5.2. Polyphenolic Compounds Analysis–Chromatographic Conditions

The Agilent 1100 HPLC Series system with UV detector, column thermostat, binary gradient pump and autosampler was employed. The HPLC system was coupled with an Agilent 1100 MS with ESI interface. A C18 RP Zorbax SB (100 × 3.0 mm, 3.5 μm) column was employed. The qualitative and quantitative analysis of polyphenols was carried out [12].The column temperature was set at 48 °C. The mobile phase was a binary gradient obtained from methanol and solution of 0.1% acetic acid (*v*/*v*). The elution started with a linear gradient for 35 minutes (from 5% to 42% methanol) and isocratic elution followed for the next 3 min (42% methanol). The solvent flow rate was maintained at 1 mL/min, the injection volume was 5 μL. The compounds’ detection was carried out on UV and MS mode. The UV detector was set at 330 nm until 17.5 min, then at 370 nm. The polyphenols eluted in less than 35 min. The collection and processing of chromatographic was done by Data Analysis and ChemStation software (Agilent Inc., Santa Clara, CA, USA).

#### 3.5.3. Mass Spectrometry Analysis 

The mass spectra signal was employed solely for identification, using the MS specific for each substance. All spectra were collected using a standard solution of polyphenols and integrated in a library. The compounds identified from MS detection were quantified by the UV trace (UV assisted by MS). The working conditions were: nitrogen temperature 350 °C at a flow rate of 12 L/min, nebulizer pressure 60 psi and capillary voltage +3000 V. The detection limits were calculated as minimal concentration producing a reproductive peak with a signal-to-noise ratio greater than three. Retention times were determined with a standard deviation ranging from 0.04 to 0.19 min. Accuracy was checked by spiking samples with a solution containing each polyphenol (10 μg/mL). 

For the quantification of polyphenolic compounds, the external standard method was employed. For the identification of polyphenols, the recorded ESI-MS and their retention times (RT) were compared to those of standards, obtained under identical conditions [38,54]. The calibration curves for a five point plot were linear in the range 0.5–50 μg/mL (R^2^ > 0.999) were further used for quantification of polyphenolic compounds in each extract.

### 3.6. Qualitative and Quantitative Analysis of Phytosterolic Compounds 

Compounds separation was done under isocratic conditions with Zorbax SB-C18 RP analytical column (100 × 3.0 mm i.d., 5 μm) and methanol:acetonitrile 10:90 (*v*/*v*) as mobile phase. The apparatus for MS analyses was Agilent Ion Trap 1100 SL MS with APCI interface, performing in positive ion mode. 

The conditions were improved in order to achieve maximum sensitivity values: nitrogen temperature 325 °C at a flow rate of 7 L/min, nebulizer pressure 60 psi and capillary voltage −4000 V.

For sterols’ identification, the RTs and MS were compared with those of standard compounds obtained under identical conditions. Instead of single ion monitoring mode (MS), the multiple reactions monitoring analysis mode was employed (MS/MS), to reduce the background’s interference. A very good linearity for calibration curves (R^2^ > 0.998) and detection limits between 59–2808 ng/mL for campesterol, 69–3312 ng/mL for ergosterol, 132–6336 ng/mL for β-sitosterol, 62–2952 ng/mL for brassicasterol and 136–6528 ng/mL for stigmasterol were obtained. 

In order to acquire and analyse the chromatographic data, Data Analysis (v5.3) and ChemStation (vA09.03) software from Agilent Inc., (USA) were used [54].

### 3.7. Qualitative and Quantitative Analysis of Iridoids 

The LC-ESI-MS/MS determination of iridoids was completed with an Agilent 1100 model coupled to an Agilent Ion Trap 1100 SL MS. An Atlantis HILIC column (100 mm × 3.0 mm, 3.5 µm) was used. A binary gradient system with eluent (A) 0.1% acetic acid and 20 µM sodium acetate in water, eluent (B) 0.1% acetic acid and 20 µM sodium acetate in acetonitrile, with the gradient of 95–80% B (0–5 min) represented the mobile phase. Data Analysis software (version B01.03, Agilent Inc., USA) was used to acquire and analyse the chromatographic data.

The MS with an ESI source worked with a scan range *m*/*z* between 360–680, in positive mode. By using the previously described LC-ESI-MS/MS method [12], the following iridoids were identified considering the adducts formed with sodium (M + 23 *m*/*z*): 8-*O*-acetylharpagide (429.3 *m*/*z*), harpagoside (517.4 *m*/*z*), harpagide (387.2 *m*/*z*), catalpol (385 *m*/*z*), aucubin (369 *m*/*z*). Their identification was confirmed by comparing the data with standards under identical chromatographic conditions. The working parameters were: drying gas flow (Nitrogen) 12 L/min, 300 °C capillary temperature and a nebulizer pressure of 60 psi. The determination coefficient for the calibration curves was R^2^ ≥ 0.990. All phytochemical analyses were done in triplicate.

### 3.8. The Evaluation of Antibacterial Activity

For the assessment of antibacterial potential, the following Gram + bacteria (*Listeria monocytogenes*, ATCC 19114, *Staphylococcus aureus*, ATCC 49444) and Gram – bacteria (*Salmonella typhimurium*, ATCC 14028, *Pseudomonas aeruginosa*, ATCC 27853, *Escherichia coli*, ATCC 25922) obtained from USAMV Cluj Napoca (Food Biotechnology Laboratory) were employed. The experiments were performed at 4 °C using Muller-Hinton Agar. 

An adjusted microdilution method was used [12,47] to determine the antibacterial effects of plants extracts. The determination of minimum inhibitory concentrations (MICs) was performed by dilution method with 96 multi-well plates. Different extract concentrations from *Ajuga genevensis* (*Ag1*) and *A. reptans* (*Ar1*) aerial parts were mixed with 10 μL of inoculum and 100 μL of Tryptic Soy Broth, followed by incubation for 24–48 h 37 °C. The lowest drug amount which could prevent the change of colour represented MIC, whereas the lowest concentration which indicates 99.5% killing of the original inoculum represents the minimum bactericidal concentration (MBC). Standard antibiotic Gentamycin (4 μg/mL, 25 μL/well) was employed as positive control, whereas 50% ethanol represented the negative control. The analyses were done three times, followed by calculation of the averages [47].

### 3.9. The Evaluation of Antifungal Activity 

Five fungi (*Penicillium funiculosum* ATCC 56755, *Candida parapsilosis* ATCC 22019, *Candida albicans* ATCC 10231, *Aspergillus niger* ATCC 6275, *Aspergillus flavus* ATCC 9643) from USAMV Cluj Napoca (Food Biotechnology Laboratory) were used to evaluate the antifungal effects of *Ajuga genevensis* (*Ag1*) and *A. reptans* (*Ar1*) extracts. The microdilution method detailed in our previous paper [12] allowed the calculation of minimum inhibitory concentration (MIC) and respectively minimum fungicidal concentration (MFC). For ethanol, chloroform and petroleum ether extracts of *Ajuga genevensis* (*Ag1*) and *A. reptans* (*Ar1*) we determined the MICs and MFCs and the positive control was Fluconazole. The experiments were repeated three times and done in duplicate [55].

### 3.10. The Evaluation of In Vivo Anti-Inflammatory Activity

#### 3.10.1. Experimental Protocol

Experimental groups consisted of Wistar albino rats obtained from the Animal Centre (Iuliu Haţieganu University of Medicine and Pharmacy Cluj-Napoca) (200–250 g). The animals (strain Crl:WI) with water ad libitum and standard pellet diet were housed prior to the experiments under a 12 h light/12 h dark cycle. 

For the evaluation of anti-inflammatory effects in vivo, 9 groups consisting of 5 animals were used, to receive the following: (1) 0.9% saline solution (1 mL, i.m. and 1 mL, i.p., negative control group), (2) turpentine oil (i.m., 6 mL/kg BW) and 0.9% saline solution (1 mL, i.p.) (inflammation group), (3) turpentine oil (i.m., 6 mL/kg BW) and 25 mg dw/mL *Ajuga genevensis* (*Ag1*) ethanol extract (5 mL/kg BW), (4) turpentine oil (i.m., 6 mL/kg BW) and 50 mg dw/mL *Ag1* ethanol extract (5 mL/kg BW), (5) turpentine oil (i.m., 6 mL/kg BW) and 100 mg dw/mL *Ag1* ethanol extract (5 mL/kg BW), (6) turpentine oil (i.m., 6 mL/kg BW) and 25 mg dw/mL *Ajuga reptans* (*Ar1*) ethanol extract (5 mL/kg BW), (7) turpentine oil (i.m., 6 mL/kg BW) and 50 mg dw/mL *Ar1* ethanol extract (5 mL/kg BW), (8) turpentine oil (i.m., 6 mL/kg BW) and 100 mg dw/mL *Ar1* ethanol extract (5 mL/kg BW), (9) turpentine oil (i.m., 6 mL/kg BW), diclofenac (20 mg/kg BW) [12,56]. 

All treatments that involved animals were rigorously in accordance with EU Directive 2010/63/EU (European guidelines and rules). The Research Ethics Committee from Iuliu Haţieganu University of Medicine and Pharmacy Cluj-Napoca approved the research protocol (No. 382/2017). 

The animals were anesthetized (20 mg/kg BW xylazine, 50 mg/kg BW ketamine), their blood collected and the obtained serum kept at −80 °C [57]. Afterwards, the rats were sacrificed by cervical dislocation [58]. 

Several parameters were determined: serum total nitrites and nitrates (NOx), total antioxidant response (TAR), oxidative stress index (OSI), total oxidative status (TOS). All experiments mentioned above were done in triplicate. 

#### 3.10.2. The Evaluation of Phagocytic Capacity

The determination of phagocytic capacity was performed as stated previously [59]. Phagocytic activity (the number of *E. coli* bacteria phagocytized by 100 leukocytes, PA) and phagocytic index (the percentage of leukocytes which phagocytized at most one bacterium, PI) were calculated. 

#### 3.10.3. The Determination of White Blood Cells (WBC) 

The WBC count was realized in a counting chamber type Bürcker-Türk, with an Olympus optical microscope. The polymorphonuclear leukocytes (PMN) and monocytes (MO) were calculated as percentage [12].

#### 3.10.4. The Assessment of Oxidative Stress 

In order to calculate the NO synthesis (NOx), we evaluated the NOx content using a Griess reaction [60], determined as μmol nitrite/L. Spectrophotometric methods were employed to determine the total oxidative status (TOS, μmol H_2_O_2_ equivalents/L) [61] and the total antioxidant response (TAR, μmol TE/L [62], whereas OSI was calculated as ratio TOS / TAR, as reported [63]. A spectrophotometer UV-VIS Jasco V-530 (Jasco International Co., Ltd., Tokyo, Japan) was employed. 

### 3.11. Statistical Analysis

The analyses were performed in triplicate and the obtained results were expressed as mean ± SD for each sample. Analysis of variance (ANOVA) was used to determine significant differences between values (*p* < 0.05), followed by multiple comparisons with Tukey test. The statistical significance of differences between extracts were evaluated by SPSS 16.0 for Windows (SPSS Inc, Chicago, IL, USA). Statistical correlations between data were calculated using the correlation analyses Pearson and Spearman.

## 4. Conclusions

In this research, two selected Romanian *Ajuga* species were evaluated for in vivo anti-inflammatory, antimicrobial and antioxidant activities, as well as for their composition in bioactive compounds. The most abundant compounds identified in the aerial parts of *Ajuga reptans* were isoquercitrin β-sitosterol and 8-*O*-acetylharpagide. The presence of these active substances may be associated with assessed biological activities. Using various methods, the antioxidant effect of *Ajuga reptans* and *Ajuga genevensis* extracts was evaluated and good antiradical capacities were evidenced, depending firstly on the nature of the extraction solvent and secondly on the different harvesting time and place. The antimicrobial assays revealed that *Ajuga reptans* petroleum ether extract presented potent activity against *A. niger* and *C. albicans*. The exhibited antifungal effects might be due to the presence of phytochemicals, primarily to 8-*O*-acetylharpagide, the main iridoid glycoside. By monitoring some inflammation parameters, it was possible to determine the in vivo anti-inflammatory activity and a probable mechanism of action could be suggested with the help of these findings. The *Ajuga reptans* 100 mg dw/mL extract displays important anti-inflammatory effects by reducing NO synthesis and it was comparable with those determined by diclofenac. The results indicate variations between the *Ajuga* species extracts, therefore, the necessity of selecting not only the proper solvent but also the appropriate harvesting time of plant material, to extract the highest possible amounts of phytochemicals. The anti-inflammatory effect of *Ajuga reptans* and *A. genevensis* ethanol extracts was observed by decreasing the oxidative stress, phagocytosis, PMN and total leukocytes. The *Ajuga reptans* 25 mg dw/mL and 100 mg dw/mL extracts presented higher anti-oxidant and anti-inflammatory activities, comparable with diclofenac. These findings support the usage of *Ajuga reptans* and *Ajuga genevensis* as anti-inflammatory agents in ethnobotanical medicine. Also, the results indicate that both selected *Ajuga* species have the potential to be valued as an important source of bioactive compounds in new herbal preparations with anti-inflammatory activity. Further in vivo experiments are recommended in order to develop effective and safe medicine-based phytopharmaceuticals.

## Figures and Tables

**Table 1:** Total phenolic content (TPC), total flavonoid content (TFC) and total iridoid content (TIC) in *A. genevensis* (*Ag1, Ag2, Ag3*) and *A. reptans* (*Ar1, Ar2, Ar3*) extracts (± SD).

Extract	TPC (mg GAE/g dw)		TFC (mg RE/g dw)		TIC (mg AE/g dw)	
	ME	EE	ME	EE	ME	EE
***Ag1***	22.59 ± 0.75	26.78 ± 0.84	15.91 ± 0.78	18.72 ± 0.85	18.44 ± 0.76	19.06 ± 0.92
***Ag2***	20.92± 0.67	23.81 ± 0.77	13.18 ± 0.69	16.27 ± 0.89	16.96 ± 0.59	17.31 ± 0.58
***Ag3***	21.95± 0.72	25.33 ± 0.81	14.07 ± 0.74	17.91± 0.73	17.52 ± 0.63	18.57 ± 0.71
***Ar1***	19.81 ± 0.87	22.97 ± 0.48	11.26 ± 0.58	14.05 ± 0.41	20.17 ± 0.91	21.03 ± 0.99
***Ar2***	17.59 ± 0.66	20.75 ± 0.72	9.98 ± 0.45	12.61 ± 0.52	18.36 ± 0.88	19.85 ± 0.86
***Ar3***	18.61 ± 0.79	21.49 ± 0.83	10.53 ± 0.64	13.19 ± 0.75	19.25 ± 0.8	20.48 ± 0.65

Note: Values are expressed as the mean ± SD (n = 3). ME—methanol extract, EE—ethanol extract.

**Table 2:** The content in polyphenolic compounds from *A. genevensis* (*Ag1*) and *A. reptans* (*Ar1*) extracts by HPLC (μg/g dw).

Polyphenolic Compound	*m*/*z* Value	Main Daughter Ions	R_T_ ± SD (min)	*A. genevensis* EE (μg/g dw)	*A. genevensis* ME (μg/g dw)	*A. reptans* EE (μg/g dw)	*A. reptans* ME (μg/g dw)
Caffeic acid	179	134.7	5.52 ± 0.09	27.88 ± 1.17	23.11 ± 1.03	-	-
*p*-Coumaric acid	163	118.7	8.7 ± 0.08	26.21 ± 1.26	18.94 ± 1.4	30.52 ± 2.29	27.81 ± 2.15
Ferulic acid	193	133.7, 148.7, 177.6	12.0 ± 0.10	18.13 ± 0.17	18.09 ± 0.25	55.73 ± 2.37	34.74 ± 2.28
Hyperoside	463	254.9, 270.9, 300.7	19.02± 0.12	6.11 ± 0.08	5.42 ± 0.13	-	-
Isoquercitrin	463	254.9, 270.9, 300.7, 342.8	19.35 ± 0.10	-	-	180.77 ± 2.84	151.1 ± 2.77
Rutin	609	254.9, 270.9, 300.7, 342.8	20.06 ± 0.15	-	-	9.67 ± 0.51	7.35 ± 0.46
Quercitrin	447	178.8, 300.7	23.44 ± 0.13	15.49 ± 0.14	11.13 ± 0.11	5.92 ± 0.43	3.46 ± 0.29
Luteolin	285	150.6, 174.6, 198.6, 240.7	29.64 ± 0.19	46.16 ± 1.93	42.97 ± 1.89	29.27 ± 0.77	28.44 ± 0.68
Apigenin	269	148.6, 150.6, 224.7, 226.7	33.10 ± 0.17	28.73 ± 1.65	25.39 ± 1.38	38.31 ± 2.28	31.89 ± 2.13

Note: Values are the mean ± SD (n = 3). ME—methanol extract, EE—ethanol extract, - Not found or below limit of detection.

**Table 3:** The content in sterols in *A. genevensis* (Ag1) and *A. reptans* (Ar1) extracts (μg/mL extract).

Extract	*β*-Sitosterol	Campesterol
***Ag1*** **EE**	-	-
***Ag1*** **PEE**	-	321.0 ± 4.39
***Ag1*** **CE**	-	832.6 ± 5.23
***Ar1*** **EE**	2048.28 ± 9.31	-
***Ar1*** **PEE**	-	511.35 ± 4.48
***Ar1*** **CE**	10923.02 ± 18.65	1446.44 ± 8.92

Note: Values are the mean ± SD (n = 3). EE—ethanol extract, PEE—petroleum ether extract, CE—chloroform extract, - Not found, below the limit of detection.

**Table 4:** The quantification of iridoids in in *A. genevensis* (*Ag1, Ag2, Ag3*) and *A. reptans* (*Ar1, Ar2, Ar3*) extracts (μg/mL extract).

Extract	Harpagide	Aucubin	Catalpol	Harpagoside	8-*O*-acetyl-harpagide
***Ag1*** **EE**	199.7 ± 4.92	8.2 ± 0.73	12.4 ± 1.68	1.2 ± 0.1	481.2 ± 5.76
***Ag1*** **ME**	193.6 ± 4.8	7.9 ± 0.68	11.7 ± 1.47	1.1 ± 0.09	475.3 ± 5.61
***Ag2*** **EE**	186.4 ± 3.71	6.1 ± 0.61	10.8 ± 1.36	0.9 ± 0.08	470.6 ± 5.44
***Ag2*** **ME**	182.5 ± 3.69	5.8 ± 0.47	10.1 ± 1.22	0.7 ± 0.05	461.9 ± 5.17
***Ag3*** **EE**	180.7 ± 3.5	5.4 ± 0.42	9.1 ± 0.85	0.8 ± 0.07	462.5 ± 5.23
***Ag3*** **ME**	177.3 ± 3.41	5.2 ± 0.39	8.8 ± 0.81	0.7 ± 0.04	460.7 ± 5.08
***Ar1*** **EE**	267.5 ± 5.74	20.7 ± 2.32	12.6 ± 1.51	0.8 ± 0.06	543.7 ± 8.61
***Ar1*** **ME**	260.2 ± 5.56	18.3 ± 2.2	11.7 ± 1.48	0.7 ± 0.06	540.8 ± 7.95
***Ar2*** **EE**	250.8 ± 5.48	18.4 ± 2.18	11.2 ± 1.44	0.5 ± 0.04	495.6 ± 7.53
***Ar2*** **ME**	248.1 ± 5.03	16.1 ± 1.97	10.3 ± 1.37	0.4 ± 0.03	492.7 ± 7.32
***Ar3*** **EE**	244.6 ± 4.98	15.9 ± 1.85	10.0 ± 1.32	0.4 ± 0.02	481.9 ± 7.07
***Ar3*** **ME**	240.3 ± 4.7	14.8 ± 1.82	9.8 ± 0.88	0.2 ± 0.01	476.5 ± 6.89

Note: ME—methanol extract, EE—ethanol extract. Values are the mean ± SD (n = 3).

**Table 5:** DPPH, TEAC, EPR activity of *A. genevensis* (*Ag1*) and *A. reptans* (*Ar1*) (mean ± SD).

Sample	DPPH IC_50_ (μg/mL) EE	DPPH IC_50_ (μg/mL) ME	TEAC mg TE/g dw	EPR mg FS/25μL	EPR mg FS/g dw
*Ag1*	31.29 ± 1.92	33.74 ± 1.99	66.13 ± 2.87	0.253 ± 0.02	94.915 ± 5.24
*Ar1*	42.75 ± 2.04	45.68 ± 2.34	60.98 ± 1.52	0.237 ± 0.01	88.896 ± 4.01
Trolox	11.2 ± 0.21				

Note: ME—methanol extract, EE—ethanol extract. Values are the mean ± SD (n = 3).

**Table 6:** Antibacterial activity of *A. genevensis* (*Ag1*) and *A. reptans* (*Ar1*) extracts.

Bacterial Strains	MIC *Ag1* (mg/mL)		MBC *Ag1* (mg/mL)		MIC *Ar1* (mg/mL)		MBC *Ar1* (mg/mL)		Gentamycin (μg/mL)	
	ME	EE	ME	EE	ME	EE	ME	EE	MIC	MBC
*S. aureus*	1.56 ± 0.01	0.78 ± 0.01	3.12 ± 0.03	1.56 ± 0.02	1.56 ± 0.01	0.78 ± 0.01	3.1 ± 0.02	1.56 ± 0.02	0.038 ± 0.001	0.076 ± 0.002
*P. aeruginosa*	3.12 ± 0.03	1.56 ± 0.02	6.25 ± 0.06	3.12 ± 0.04	3.12 ± 0.02	1.56 ± 0.01	6.25 ± 0.07	3.12 ± 0.02	1.2 ± 0.02	2.4 ± 0.04
*L. monocytogenes*	6.25 ± 0.08	6.25 ± 0.07	12.5 ± 0.09	12.5 ± 0.09	6.25 ± 0.08	3.12 ± 0.03	12.5 ± 0.09	6.25 ± 0.07	0.076 ± 0.001	0.15 ± 0.01
*E. coli*	6.25 ± 0.07	3.12 ± 0.03	12.5 ± 0.07	6.25 ± 0.08	6.25 ± 0.07	3.12 ± 0.04	12.5 ± 0.08	6.25 ± 0.06	1.2 ± 0.01	2.4 ± 0.05
*S. typhimurium*	6.25 ± 0.09	6.25 ± 0.08	12.5 ± 0.09	12.5 ± 0.09	6.25 ± 0.08	3.12 ± 0.03	12.5 ± 0.09	6.25 ± 0.08	2.4 ± 0.03	4.8 ± 0.07

Note: ME—methanol extract, EE—ethanol extract.

**Table 7:** Antifungal activity of *A. genevensis* (*Ag1*) and *A. reptans* (*Ar1*) extracts.

Bacterial Strains	MIC *Ag1* (mg/mL)			MFC *Ag1* (mg/mL)			MIC *Ar1* (mg/mL)			MFC *Ar1* (mg/mL)			Fluconazole (μg/mL)	
	EE	PEE	CE	EE	PEE	CE	EE	PEE	CE	EE	PEE	CE	MIC (μg/mL)	MFC (μg/mL)
*Aspergillus flavus*	0.05 ± 0.008	0.012 ± 0.003	0.025 ± 0.007	0.1 ± 0.03	0.025 ± 0.006	0.05 ± 0.008	0.025 ± 0.006	0.12 ± 0.04	0.006 ± 0.0003	0.05 ± 0.008	0.25 ± 0.04	0.012 ± 0.005	0.15 ± 0.03	0.3 ± 0.05
*Aspergillus niger*	0.1 ± 0.02	0.012 ± 0.005	0.012 ± 0.004	0.2 ± 0.06	0.025 ± 0.005	0.025 ± 0.006	0.05 ± 0.008	0.012 ± 0.005	0.012 ± 0.004	0.1 ± 0.03	0.025 ± 0.005	0.025 ± 0.006	0.15 ± 0.03	0.3 ± 0.06
*Candida albicans*	0.025 ± 0.006	0.012 ± 0.006	0.025 ± 0.007	0.05 ± 0.008	0.025 ± 0.005	0.05 ± 0.008	0.012 ± 0.005	0.006 ± 0.0003	0.012 ± 0.004	0.025 ± 0.006	0.012 ± 0.005	0.025 ± 0.007	0.1 ± 0.02	0.2 ± 0.04
*Candida parapsilosis*	0.025 ± 0.006	0.012 ± 0.005	0.05 ± 0.009	0.05 ± 0.008	0.025 ± 0.007	0.05 ± 0.008	0.025 ± 0.007	0.025 ± 0.006	0.012 ± 0.003	0.05 ± 0.009	0.05 ± 0.008	0.025 ± 0.005	0.1 ± 0.02	0.2 ± 0.04
*Penicillium fumiculosum*	0.1 ± 0.03	0.05 ± 0.008	0.025 ± 0.006	0.2 ± 0.05	0.1 ± 0.03	0.05 ± 0.009	0.05 ± 0.008	0.05 ± 0.007	0.05 ± 0.008	0.1 ± 0.02	0.1 ± 0.03	0.1 ± 0.02	0.15 ± 0.03	0.3 ± 0.06

Note: PEE—petroleum ether extract, EE—ethanol extract, CE—chloroform extract.

**Table 8:** Anti-inflammatory activity of *A. genevensis* (*Ag1*) and *A. reptans* (*Ar1*) extracts (WBC, PMN, MO, PA, PI, TAR, TOS, NO, OSI).

Parameter	*Ag* 100 mg dw/mL	*Ag* 50 mg dw/mL	*Ag* 25 mg dw/mL	*Ar* 100 mg dw/mL	*Ar* 50 mg dw/mL	*Ar* 25 mg dw/mL	Inflam	Diclo
**WBC**	5953.2 ± 325.22	10077.8 ± 394.84	5086.6 ± 419.96	4860 ± 669.21	6884 ± 413.55	5030 ± 242.07	11602 ± 649.63	4866.8 ± 581.31
**PMN**	70.6 ± 5.27	71.4 ± 6.06	59.2 ± 4.38	59.6 ± 2.96	62 ± 7.31	61.4 ± 3.43	79.6 ± 2.6	54.4 ± 3.84
**MO**	2.6 ± 0.89	2.6 ± 0.89	2.2 ± 0.44	2.4 ± 0.89	2.6 ± 0.89	2.6 ± 0.89	2.8 ± 1.09	2.4 ± 0.89
**PA**	26.4 ± 3.57	49.6 ± 1.67	28 ± 3.46	21.2 ± 2.68	34.4 ± 2.6	23.6 ± 1.67	50.4 ± 2.19	23.8 ± 1.78
**PI**	52.8 ± 3.89	86.8 ± 5.4	40.8 ± 2.28	27.2 ± 2.28	32 ± 4.24	30.8 ± 2.28	86.8 ± 4.14	37.2 ± 3.03
**TAR**	1.0890 ± 0.003	1.0857 ± 0.0002	1.0852 ± 0.0003	1.0866 ± 0.0003	1.0871 ± 0.001	1.0862 ± 0.0004	1.0871 ± 0.0008	1.0894 ± 0.0006
**TOS**	16.602 ± 1.22	23.095 ± 4.25	20.461 ± 4.21	15.767 ± 1.66	19.913 ± 5.43	18.584 ± 1.35	22.469 ± 2.64	16.208 ± 0.85
**NO**	57.113 ± 4.85	49.219 ± 7.21	57.083 ± 2.13	43.770 ± 5.03	49.631 ± 8.2	59.322 ± 4.08	59.293 ± 5.07	33.231 ± 7.35
**OSI**	15.247 ± 1.61	21.270 ± 3.91	18.854 ± 3.88	14.510 ± 1.53	18.314 ± 4.97	17.109 ± 1.25	20.667 ± 2.42	14.878 ± 0.78

**Note:***Ag* 100 mg/mL—animals were given 5 mL/kg BW *A. genevensis* ethanol extract 100 mg dw/mL, *Ag* 50 mg/mL—animals were given 5 mL/kg BW *A. genevensis* ethanol extract 50 mg dw/mL, *Ag* 25 mg/mL—animals were given 5 mL/kg BW *A. genevensis* ethanol extract 25 mg dw/mL, *Ar* 100 mg/mL—animals were given 5 mL/kg BW *A. reptans* ethanol extract 100 mg dw/mL, *Ar* 50 mg/mL—animals were given 5 mL/kg BW *A. reptans* ethanol extract 50 mg dw/mL, *Ar* 25 mg/mL—animals were given 5 mL/kg BW *A. reptans* ethanol extract 25 mg dw/mL, Inflam—the induction of inflammation was made by intramuscular injection of turpentine oil (6 mL/kg BW), Diclo—animals were given 20 mg/kg BW diclofenac.
